# The Prognostic Scoring System Establishment and Validation for Chronic Atrial Fibrillation Patients Receiving Modified Cox-Maze IV and Concomitant Cardiac Surgery

**DOI:** 10.1371/journal.pone.0126300

**Published:** 2015-06-11

**Authors:** Feng-Chun Tsai, Heng-Tsan Ho, Jen-Ping Chang, Feng-Chang Tsai, Jaw-Ji Chu, Pyng-Jing Lin

**Affiliations:** 1 Department of Cardiothoracic and Vascular Surgery, Chang Gung Memorial Hospital, Linkou, Taiwan; 2 Chang Gung University of Medicine, Kwei-Shan, Taoyuan, Taiwan; 3 Chang Gung University College of Medicine, Kwei-Shan, Taoyuan, Taiwan; 4 Department of Cardiothoracic and Vascular Surgery, Chang Gung Memorial Hospital, Kaohsiung, Taiwan; 5 Formosa Biomedical Technology, Chang Gung Memorial Hospital, and Chang Gung University, Taoyuan, Taiwan; Sapienza University of Rome, ITALY

## Abstract

**Objectives:**

Traditional Cox maze III is the gold standard for treatment of atrial fibrillation (AF). Because of its invasiveness, it has been replaced by a simplified procedure involving radiofrequency ablation of modified Cox maze IV. Although the modified Cox maze IV has the advantages of simplicity and less morbidity, a lower rate of sinus rhythm conversion has been reported. We try to establish a scoring system to predict the outcome of this procedure.

**Methods and Results:**

The derivation group consisted of 287 patients with structural heart disease and chronic AF who underwent cardiac surgery and modified Cox-maze IV procedure between August 2005 and March 2013. Demographics, clinical and laboratory variables were retrospectively collected as sinus conversional predictors. Overall sinus conversion rate was 75.8%. The parameters of the Soft Markers Scoring system included AF duration, preoperative left atrial (LA) size, rheumatic pathology and postoperative LA remodeling. We compared 80 patients from another hospital between January 2004 and December 2011 as a validation group to evaluate the power of the scoring system. Soft Markers Score indicated a good discriminative power by using the areas under the receiver operating characteristic curve (AUROC: 0.759 ± 0.032). The score was further divided into three groups: low (0-2), intermediate (3-5), and high (6-10), with predicted sinus conversion rates of 92.4%, 74.2%, and 47.8%, respectively.

**Conclusions:**

In patients with chronic AF receiving modified Cox-maze IV procedure, the Soft Markers Score demonstrated good discriminative power of predicting sinus recovery in our patients and applied well to the other validation populations.

## Introduction

The gold standard treatment for atrial fibrillation (AF), Cox-Maze III procedure, successfully restores atrio-ventricular synchrony and decreases the risk of thromboembolism and stroke. [1] Because of its invasiveness, it has been replaced in most clinical practice by a simplified procedure involving radiofrequency (RF) ablation, modified Cox maze IV. Although the modified Cox maze IV has the advantages of feasibility and decreased morbidity, a lower rate of sinus rhythm recovery has been reported compared with the maze III. [2,3] According to the previous reports, preoperative left atrial (LA) size and the duration of AF are the two most important predictors of sinus conversion. [4–6] However, most reports did not incorporate postoperative LA remodeling to predict the outcome. Collecting both preoperative and postoperative parameters to establish a scoring system to predict the procedure success may be an attractive alternative. In this retrospective study, we examined the Soft Markers Score and other predictors for patients with structural heart disease and chronic AF who underwent concomitant cardiac surgery and modified Cox-Maze IV procedure, aiming to identify the relationship between the sinus rhythm recovery rate and the prognostic scoring system. Moreover, we compared with the validation group from another hospital to confirm the power of the Soft Markers Score.

## Materials And Methods

### Study population

This retrospective study was conducted after receiving the approval of the Institutional Review Board (IRB) both from our hospital, Chang Gung Memorial Hospital, LinKou, and the other hospital, Chang Gung Memorial Hospital, Kaohsiung, of the validation group (approval No. 102-4227B). Written informed consent for the AF ablation procedure was collected from all patients prior to surgery. The enrolled and excluded criteria in validation group were the same with the derivation group. Only patients with structural heart disease receiving concomitant cardiac surgery were enrolled. Lone AF was excluded from this study. With the aid of a RF device, AF ablation became a routine adjunct operation if patients were willing to receive the procedure and if it was not contraindicated, such as in cases of significant calcification of the left atrial wall in extreme rheumatic heart disease or redo cases with severe pericardial adhesion. The derivation group of 287 patients was collected between August 2005 and March 2013 and compared with the validation group of 80 patients who were operated on between January 2004 and December 2011.

### Surgical procedure

**Fig 1:**
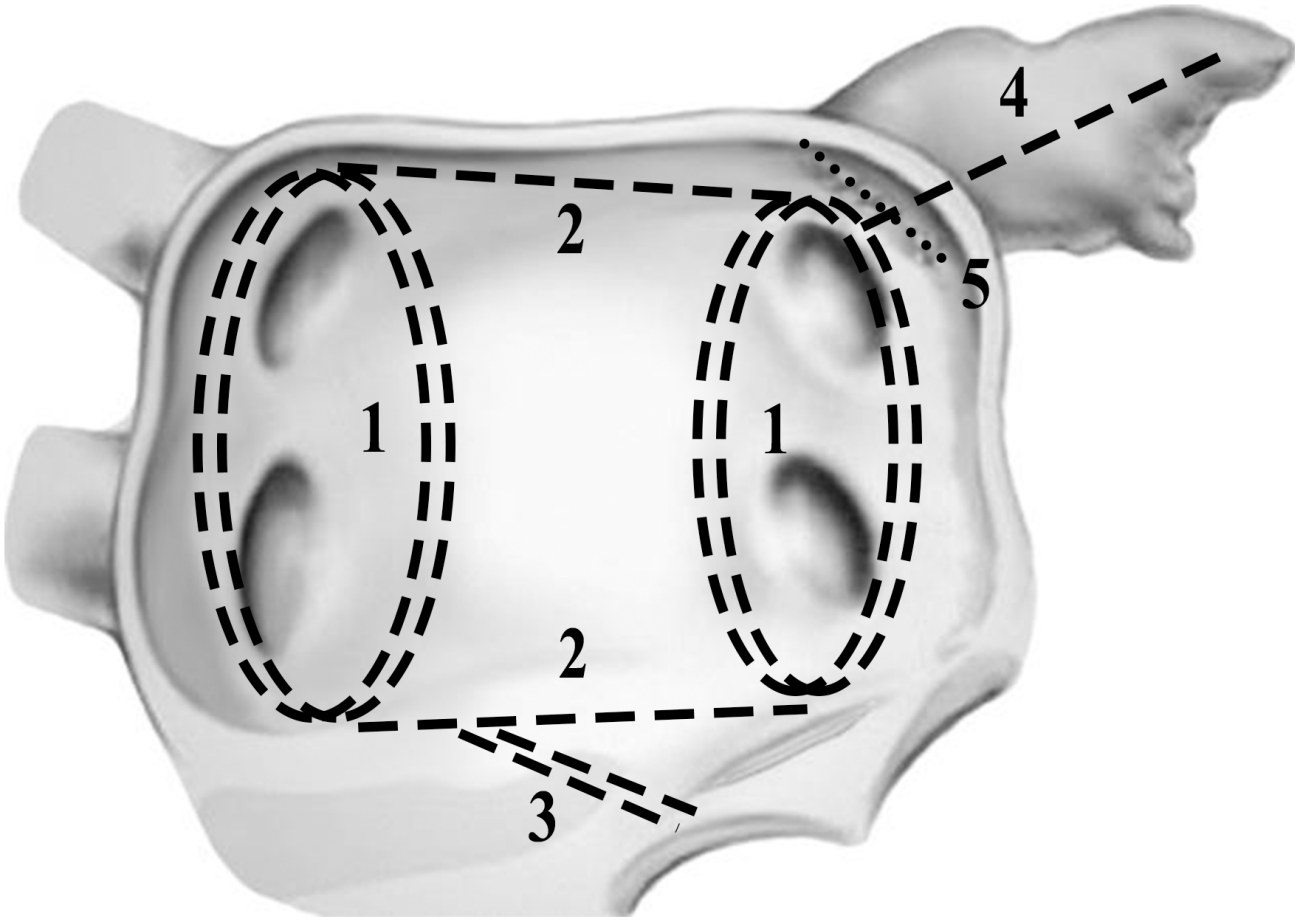
Lesion set of the modified Cox-maze IV procedure, Left atrium. 1. Pulmonary veins isolation: Twice under beating heart. 2. Connect bilateral PV: Double line. 3. Connect to mitral annulus: Twice. 4. Connect LAA to LPV. 5. Suture closure of LAA: From interior, double layers. Additional procedures: divide ligament of Marshall; LA volume reduction. (PV: Pulmonary vein, LAA: Left atrial appendix, LPV: Left pulmonary vein, LA: Left atrium)

**Fig 2:**
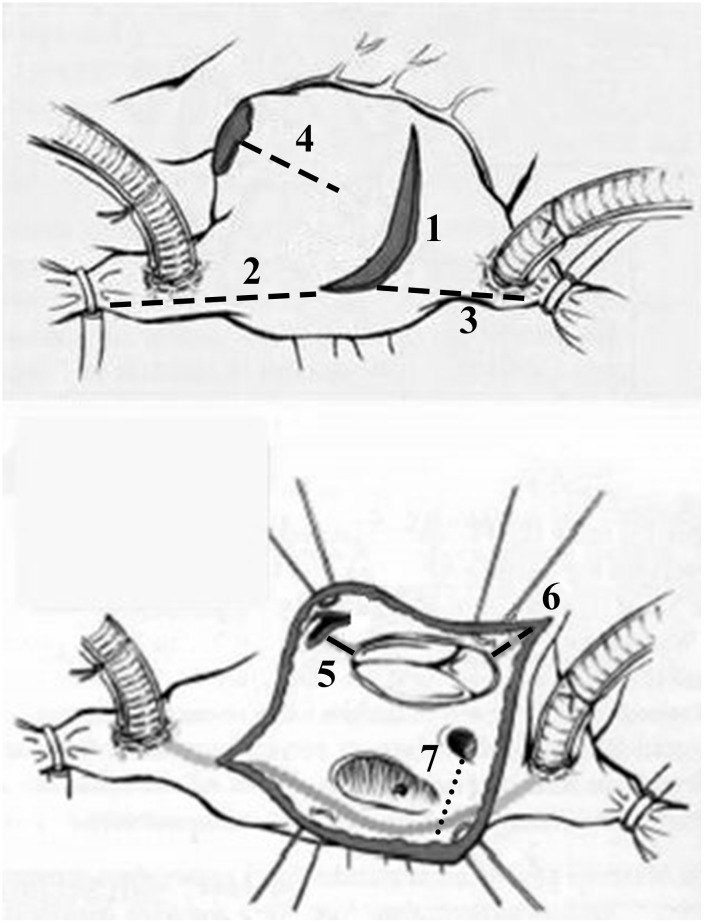
Lesion set of the modified Cox-maze IV procedure, Right atrium. 1. Prepare: transverse incision, amputate RAA, loop RAA &RA atriotomy with silk for exposure, free AV groove & RA dome. 2. Connect incision to SVC, beware of SA Node & swan ganz catheter. 3. Connect incision to IVC. 4. Connect from RAA to free wall, leave 2 cm gap. 5. Connect to 10” o’clock of TV annulus. 6. Connect incision to 4” o’clock of TV annulus. 7. Ablation of coronary sinus from inside. (RAA: Right atrial appendix, RA: Right atrium, SVC: Superior vena cava, IVC: Inferior vena cava, TV: Tricuspid valve)

Patients of the derivation group were operated on by three senior surgeons with technical unity and the first ten cases were excluded to avoid learning curve bias. Our routine approach was standard full sternotomy with bicaval cannulations, and AF ablation lesion sets were similar to traditional Cox-Maze III except most cut-and-sew lesions were replaced by the RF ablation device and separated pulmonary vein isolation with two connecting lesions of both superior and inferior pulmonary veins. [7] The complete lesion sets were as the Fig 1 and Fig 2. Additional cryoablation of mitral isthmus was applied with cryoprobe for 2 minutes. Bi-atrial ablation was applied in most cases and left atrial ablation alone was applied solely in elderly patients with isolated aortic valve replacement. The left atrial appendage was closed from inside with 4–0 prolene double running sutures. The ligament of Marshall was divided during left pulmonary vein isolation and bilateral epicardial fats over the interatrial groove, comprised of ganglional plexi, were also resected. Left atrial volume reduction for markedly enlarged left atrium, dimensions > 60 mm by preoperative echo, was carried out with plications of posterior wall between the inferior pulmonary vein and mitral isthmus.

### Postoperative medications and follow-up

Class III antiarrhythmic agent, Amiodarone, was used if AF remained postoperatively and without contraindications of junctional bradycardia or prolonged QT interval. After 3 months, if AF persisted, beta blockers were prescribed instead of anti-arrhythmics. Anticoagulant was discontinued at 3 months for patients with sinus conversion and without other lifelong mandatory indications such as receiving mechanical valve replacement. Both of the preoperative and postoperative cardiac measurements were recorded by transthoracic echocardiography. The left atrial diameter was measured in the parasternal long-axis view from the trailing edge of the posterior aortic root-anterior left atrial complex to the posterior left atrial wall at end-systolic phase. The twelve leads electrocardiograms (EKG) were checked monthly at the first three months and then every two to three months periodically at outpatient clinic visit to document atrial rhythm. No routine 24 hours Holter monitoring or event recorder was used unless patients complained of paroxysmal palpitations while resting EKG showed sinus rhythm. Failure of sinus conversion was defined as detection of an episode of AF, atrial flutter or atrial tachycardia lasting more than 30 seconds by Holter. At least one-year follow up was completed for every patient.

### Establishment of Soft Markers Scoring system and Validation

Each risk factor in the derivation group was recognized by multivariate logistic regression and the cutoff point of parameters was calculated by acquiring the best Youden Index (sensitivity + specificity -1). While establishing Soft Markers Score parameters from the univariate analysis, we found that rheumatic heart disease had a wider confidence interval compared with other risk factors. We speculated this factor would represent lower power of prediction in the scoring system and defined it as a minor parameter of one score point. The other risk factors defined as intermediate and major parameters were two and three score points, respectively. This comprised of our scoring system with one minor parameter, rheumatic heart disease, three intermediate parameters including preoperative LA diameter over 60 mm, postoperative LA diameter over 50 mm and reduced LA diameter less than 10 mm in echocardiography and one major parameter of preoperative AF duration (Table 1). To test the Soft Markers Score as a powerful independent predictor of the sinus recovery in chronic AF patients, we compared the areas under the receiver operating characteristic (AUROC) curve of the Soft Markers Score both in the derivation and validation group.

**Table 1:** Soft Marker Scoring system.

Point	Parameter
1 point	Rheumatic heart disease
2 points	Preoperative LA diameter > 60 mm
	Postoperative LA diameter > 50 mm
	Postoperative LA reduction < 10 mm
3 points	AF duration > 4 years

LA: left atrial

### Statistical analysis

Descriptive statistics such as number of observations, mean and standard deviation will be presented for continuous variables. The primary analysis compared rates of sinus recovery with whom of persistent AF at the last follow up. All variables were tested for normal distribution using the Kolmogorov-Smirnov test. The Student’s t test was utilized to compare the means of continuous variables and normally distributed data. If assumption of normality is not satisfied, the use non-parametric analysis, the Mann-Whitney U test, will be considered. Categorical data were tested with the chi-square test or the Fisher exact test. Risk factors for sinus conversion were assessed first by univariate logistic regression, and enrolled into a multivariate analysis if statistical significance was met (*p* < 0.05). The multivariate analyses were assessed by applying multiple logistic regressions based on forward data eliminations. Calibration was assessed using the Hosmer-Lemeshow goodness-of-fit test (C statistic) to compare the number of observed abnormal cardiac rhythms. Discrimination was assessed using the AUROC curve. Areas under two AUROC curves were compared with a non-parametric approach. The AUROC analysis was also performed to calculate cutoff values, sensitivity, and specificity. All statistical tests were two- tailed and evaluated at 0.05 level of significance. Data were analyzed using SPSS 20.0 for Windows (SPSS, Inc., Chicago, IL, USA) and STATA 11.0 for Mac (StataCorp LP, Texas, USA).

## Result

### Patients’ demographics

From August 2005 to March 2013, 287 patients (age: 20.3–85.6; mean: 59.0±0.7 years old) underwent the modified Cox-maze IV ablation of AF in addition to cardiac surgery. Concomitant procedures included mitral valve replacement (n = 95), mitral repair (n = 162), aortic valve replacement (n = 53), tricuspid repair (n = 159), and coronary artery bypass graft (n = 23). There were 4.9% (n = 14) hospital mortality and follow-up was completed at 99.3% (n = 285) with a mean follow-up of 38 months (range 12–96), at least twelve months for every patient. Less than 5% of the patients underwent Holter examinations. Final sinus conversion rate without any antiarrhythmic medication was 75.8% (n = 216). Demographics and clinical characteristics of both sinus recovery and non-sinus conversion group were listed in Table 2 and different mitral valve pathology related to sinus conversion was presented in Table 3.

**Table 2:** Patients’ demographics and clinical characteristics according to sinus recovery

		Postoperative rhythm		
	All patient (n = 287)	Sinus (n = 216)	Non-sinus (n = 71)	*p*
Age (years)	59.0 ± 0.7	58.6 ± 0.8	60.3 ± 1.0	0.203
Gender, male	140 (48.8%)	108 (50%)	32 (45.1%)	0.471
BMI (kg/m2)	23.4 ± 0.2	23.5 ± 0.3	23.2 ± 0.4	0.494
DM	39 (13.6%)	27 (12.5%)	12 (16.9%)	0.348
ESRD	5 (1.7%)	4 (1.9%)	1 (1.4%)	1.000
Serum creatinine (mg/dL)	1.09 ± 0.08	1.14 ± 0.10	0.90 ± 0.03	0.512
AF duration (month)	53.9 ± 3.7	44.9 ± 4.5	75.4 ± 5.3	< 0.001
PA systolic pressure (mmHg)	55.9 ± 1.3	55.2 ± 1.5	57.6 ± 2.3	0.344
PA diastolic pressure (mmHg)	27.1 ± 0.6	27.3 ± 0.7	26.8 ± 1.2	0.739
PA mean pressure (mmHg)	38.5 ± 0.8	38.4 ± 1.0	38.8 ± 1.4	0.685
CHADS2 score	1.5 ± 0.1	1.5 ± 0.1	1.4 ± 0.1	0.446
Bi-atrial ablation	267 (93.0%)	200 (92.7%)	67 (94.4%)	0.426
LA reduction	130 (45.3%)	90 (41.7%)	40 (56.3%)	0.031
Mitral valve repair/replacement	276 (96.2%)	208 (96.3%)	68 (95.8%)	0.736
Tricuspid valve repair	159 (55.4%)	111 (51.4%)	48 (67.6%)	0.017
Aortic valve replacement	53 (18.5%)	36 (16.7%)	17 (23.9%)	0.170
CABG	23 (8.0%)	21 (9.7%)	2 (2.8%)	0.063
Complex procedure*	49 (17.1%)	38 (17.6%)	11 (15.5%)	0.683
Numbers of valve surgeries	1.7 ± 0	1.7 ± 0	1.9 ± 0.1	0.008
Leaving OR rhythm, sinus	161 (71.9%)	123 (72.4%)	38 (70.4%)	0.778
Need mechanical support**	13 (4.5%)	7 (3.2%)	6 (8.5%)	0.095
Need temporary pacing	51 (17.8%)	41 (19.1%)	10 (14.1%)	0.341
Pre OP EF %	59.5 ± 0.8	58.2 ± 1.0	63.5 ± 1.2	0.014
Pre-OP LA diameter (mm)	58.9 ± 0.7	57.4 ± 0.7	63.4 ± 1.4	< 0.001
Pre-OP LVEDD (mm)	55.1 ± 0.6	55.6 ± 0.8	53.4 ± 1.0	0.092
Pre-OP LVESD (mm)	36.9 ± 0.6	37.7 ± 0.8	34.2 ± 0.9	0.003
Post-OP EF %	60.8 ± 0.8	60.7 ± 1.0	61.1 ± 1.2	0.649
Post-OP LA diameter (mm)	47.2 ± 0.6	45.5 ± 0.7	52.1 ± 1.1	< 0.001
Post-OP LVEDD (mm)	48.8 ± 0.5	48.9 ± 0.6	48.5 ± 0.8	0.663
Post-OP LVESD (mm)	32.5 ± 0.6	32.6 ± 0.7	32.3 ± 0.8	0.421
Post-OP LA reduced diameter (mm)	11.9 ± 0.7	11.5 ± 0.8	11.0 ± 1.4	0.785
Soft Marker Score	2.8 ± 0.1	3.2 ± 0.2	5.5 ± 0.3	< 0.001

AF: atrial fibrillation; BMI: body mass index; CABG: coronary artery bypass graft; DM: diabetes mellitus; EF: ejection fraction; ESRD: end stage renal disease; LVEDD: left ventricle end-diastolic diameter; LVESD: left ventricle end-systolic diameter; OP: operation; OR: operation room; PA: pulmonary artery

*Complex procedure: combined coronary artery bypass or triple valve surgeries.

**Mechanical support: Intra-aortic balloon pump (IABP) or extracorporeal membrane oxygenation (ECMO)

**Table 3:** Mitral valve pathology data according to sinus recovery

		Postoperative rhythm		
	All patient (n = 265)	Sinus (n = 199)	Non-sinus (n = 66)	*p*
Degenerative	91 (34.3%)	71 (35.7%)	20 (30.3%)	0.434
Ischemic	21 (7.9%)	17 (8.5%)	4 (6.1%)	0.518
Rheumatic	112 (42.3%)	76 (38.2%)	36 (54.5%)	0.020
Dilated	35 (13.2%)	30 (15.1%)	5 (7.6%)	0.119
Endocarditis	3 (1.1%)	3 (1.5%)	0 (0%)	0.576
Previous prosthesis dysfunction*	10 (3.8%)	6 (3.0%)	4 (6.1%)	0.272

*: bioprosthesis degeneration/endocarditis or mechanical valve thrombosis/endocarditis

### Calibration and Discrimination for Risk Factor and Soft Markers Score

Hosmer-Lemeshow chi-square statistic of predicted non-sinus recovery risk and the AUROC were used to assess calibration and discrimination, respectively. Table 4 compares some important predictors of sinus conversion for these patients. Calibration for Soft Markers Score (Hosmer-Lemeshow chi-square 6 = 5.409; *p* = 0.493) was good. The AUROC curve confirmed the good discrimination power of the Soft Markers Score (AUROC, 0.759 ± 0.032; 95% confidence interval [CI], 0.695–0.822, *p* < 0.001) compared with preoperative, postoperative LA diameter, and AF duration.

**Table 4:** Comparison of calibration and discrimination of the score and risk factors in sinus recovery

	Calibration			Discrimination		
	Hosmer-Lemeshow	df	*p*	AUROC ± SE	95% CI	*p*
Soft Marker Score	5.409	6	0.493	0.759 ± 0.032	0.695–0.822	< 0.001
AF duration	16.811	8	0.032	0.708 ± 0.041	0.626–0.789	< 0.001
Pre-OP LA diameter	5.296	8	0.726	0.650 ± 0.037	0.579–0.722	< 0.001
Post-OP LA diameter	14.369	8	0.073	0.711 ± 0.035	0.642–0.780	< 0.001

### Long-Term Prognosis and Validation

**Fig 3:**
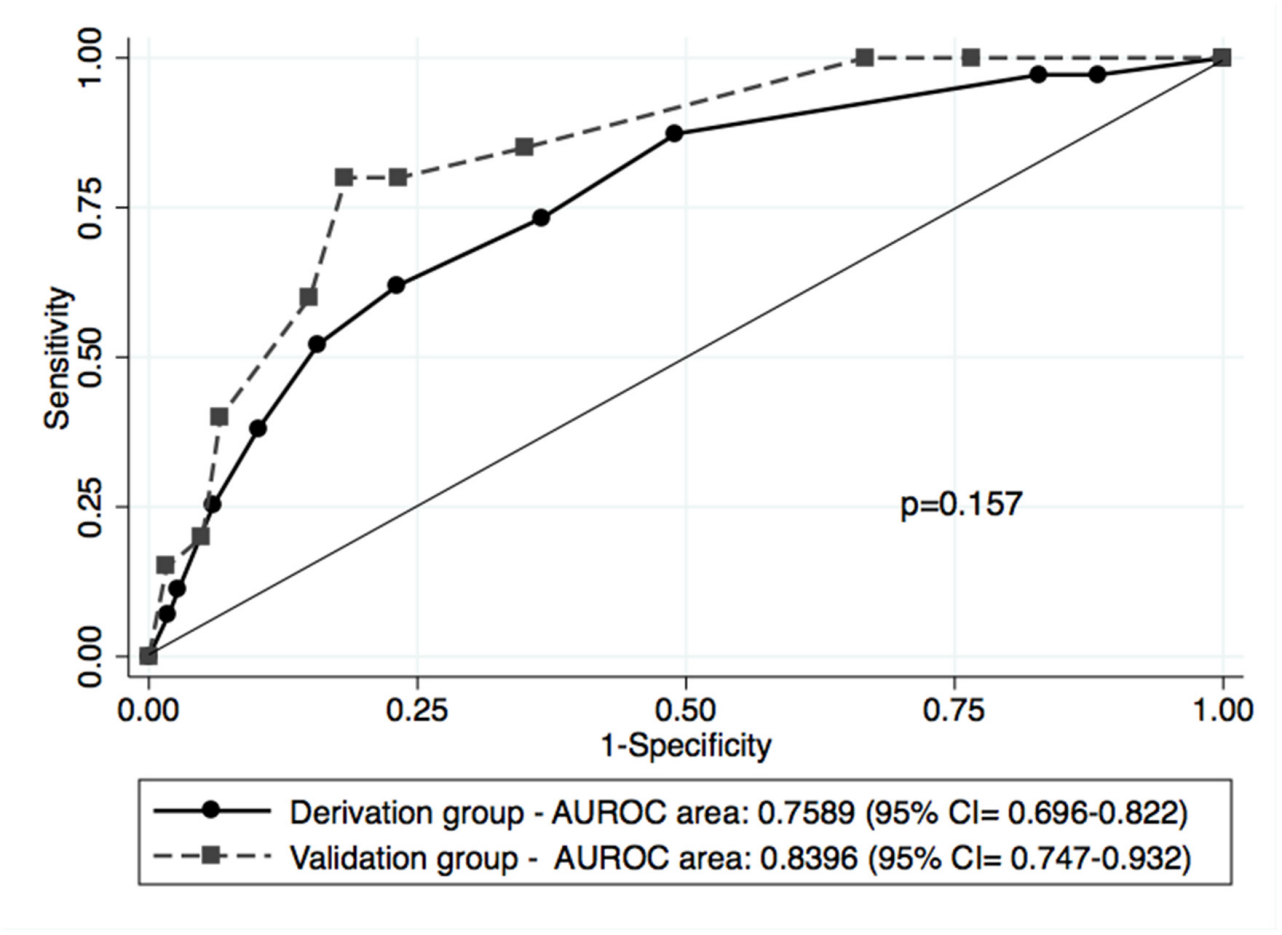
AUROC curve of Derivation and Validation groups. This is the Soft Marker Score ROC curve of the derivation and validation groups. It shows the good discrimination power of the Soft Marker Score and the p-value shows there was no statistically significant difference between the derivation and validation group. (CI: confidence interval)

In multivariate analysis without the Soft Markers Score, the preoperative LA size and postoperative LA size were all the independent predictors. After we included the Soft Markers Score, though several variables showing prognostic significance in univariate analysis, only Soft Markers Score was identified as independent predictor in multivariate analysis in Table 5. The logarithm of odds = -2.314 + 0.46 × Soft Markers Score. Table 6 lists the demographics, medical histories, and outcomes in the derivation and validation groups. We found the patients in the validation group had shorter AF duration, smaller LA diameters and better EF before operation, and more reduced LA diameter after operation. A comparison of the AUROC between derivation group and validation group confirms the good discrimination power of the Soft Marker Score in the validation group (AUROC, 0.840 ± 0.049; 95% confidence interval [CI], 0.747–0.932, *p* < 0.001). Moreover, the nonparametric comparison revealed that there was no statistically significant difference between the derivation and the validation group (Fig 3). The Soft Marker Score provided good predictor power in both groups and was divided into 3 categories: Low, intermediate and high risks. The low risk group, compromising scores from 0 to 2, intermediate risk group of 3 to 5 and high risk group of 6 to 10 points had sinus rhythm recovery rates of 92.4%, 74.2%, and 47.8%, respectively. The overall sinus recovery rate in the Soft Markers Score is shown in Table 7.

**Table 5:** Variables showing prognostic significance

	Univariate logistic regression		Multivariate logistic regression	
Parameter	OR (95% CI)	*p*	OR (95% CI)	*p*
AF duration (month)	1.013 (1.006–1.020)	< 0.001		
LA reduction	1.806 (1.051–3.104)	0.032		
Tricuspid valve repair	1.974 (1.123–3.470)	0.018		
Numbers of valve surgeries	1.766 (1.146–2.719)	0.010		
Pre OP EF %	1.031 (1.088–1.054)	0.007		
Pre-OP LA diameter (mm)	1.049 (1.023–1.076)	< 0.001		
Pre-OP LVESD (mm)	0.964 (0.936–0.993)	0.015		
Post-OP LA diameter (mm)	1.083 (1.047–1.119)	< 0.001		
Mitral: rheumatic pathology	1.942 (1.106–3.409)	0.021		
Soft Marker Score	1.444 (1.285–1.624)	< 0.001	1.452 (1.253–1.683)	< 0.001

**Table 6:** Patients’ demography data in Derivation and Validation groups

	Derivation (n = 287)	Validation (n = 80)	*p*
**Demographics**			
Age (years)	59.0 ± 0.7	56.7 ± 1.5	0.119
Gender, male	140 (48.8%)	38 (47.5%)	0.839
BMI (kg/m^2^)	23.4 ± 0.2	23.1 ± 0.4	0.504
**Medical history**			
AF duration (months)*	53.9 ± 3.7	51.8 ± 7.4	0.049
Pre OP LA diameter (mm)	58.9 ± 0.7	54.4 ± 1.1	0.001
Pre OP EF%	59.5 ± 0.8	64.6 ± 1.4	0.004
Bi-atrial ablation	224 (78.0%)	67 (83.8%)	0.073
Mitral valve surgery	276 (96.2%)	78 (97.5%)	0.742
Rheumatic heart disease	112 (42.3%)	35 (43.8%)	0.814
**Outcome**			
Sinus conversion	216 (75.3%)	60 (75.0%)	0.998
Post OP LA diameter	47.2 ± 0.6	43.1 ± 1.1	0.001

*Compared with patient whose AF durations were known

**Table 7:** Sinus recovery rate in Soft Marker Score

Soft marker score	Sinus recovery rate
Low risk (score 0–2)	92.4%
Intermediate risk (score 3–5)	74.2%
High risk (score 6–10)	47.8%

## Discussion

Modified Cox maze IV with RF assisted ablation has replaced the traditional cut and sew in most clinical practice because of its simplicity and less invasiveness. However, less sinus conversion drives us to pursue a better tool to predict the outcome of surgical ablation, focus more on potentially correctable factors and improve the success rate [2,3]. The Soft Markers Score that we established here comprised of four important parameters: duration of AF, preoperative LA size, pathology of the mitral valve and the postoperative LA remodeling.

The duration of AF and the size of LA were the most independent prognostic predictors for patients underwent the Cox-maze procedure. [4–6] In spite of these two factors may influence the outcome independently, they are reciprocal causation. [8] The AF would induce atrial dysfunction, which contributed to myocardial constriction function impairment and chamber dilatation. Dilatation of the atrial wall would break the normal structure of the conducting system, trigger the macro-reentry development, making the AF more persistent and developing a vicious cycle of AF begets AF. From our previous reports and others, preoperative LA size was significantly larger in patients who experienced recurrent AF after modified Cox-maze IV procedure. [5,9–11] Some further used the LA volume index or left atrial emptying fraction to predict the outcome of the operation. [12,13]

In contrast to the previous report that pathology of mitral lesion was not a predictor of AF recurrence, [14] we found that chronic AF patients associated with rheumatic etiologies had lower sinus conversion rates when compared with other mitral pathologies. These attributes of a significant predictor may echo the hypothesis that atrial extracellular matrix alternation or atrial wall fibrosis could play a role in AF maintenance. [15,16] More studies may be necessary to determine whether the lower sinus conversion rate was really caused by this particular pathology alone or just a coincidental result of the association between larger LA size and prolonged AF duration.

Not only preoperative LA size and function are important to the prognosis of modified Cox-maze IV procedure, the postoperative LA size and remodeling may also influence the outcome. [17] Structural remodeling of the pulmonary veins and LA can be reversible after successful catheter ablation without AF recurrence; however, late recurrence of AF is associated with progressive LA dilatation. [18] The LA size decreased during follow-up in patients with sustained sinus rhythm, whereas LA size increased in cases of recurrent AF.[4] This raises the tempting possibility that reducing atrial size may help to mitigate the re-entry circuits underlying AF, thus increasing the success rate of modified Cox-maze IV procedure. However, the evidence is not strong enough to draw solid conclusions since it was not randomized and cannot be generalized, owing to substantial variations in the populations. [19]

Despite the promising results of this study, several important limitations must be recognized. First, though duration of the AF was the most important parameter in our scoring system, its duration may be under estimated. We can only count it until the patient’s presentation to the hospital. Second, some missing details of data collection should be expected in this retrospective study such as the pulmonary arterial pressure or New York Heart Association functional class during the whole time span. Third, postoperative echocardiography was not performed with a tight time frame and by a core lab examiner. Therefore, the size of LA measurement may show some variations. Finally but not last, we applied our score system to the validation group from another hospital with acceptable outcome. Without propensity matched population because of the limitation of numbers, the pre-existing differences in patients’ demographics between the two groups may compromise the outcome and jeopardize the final judgment. Cooperation with multi-centers, prospective data collection and adhering to strict echo follow-up protocol with a core lab may minimize the bias and confirm the accuracy of this scoring system.

In conclusion, patients with structural heart disease combined with permanent AF who underwent cardiac surgery and concomitant modified Cox-maze IV procedure attained a sinus rhythm recovery rate of 75.8% in this study. The Soft Markers Score, including four important parameters of duration of AF, preoperative LA size, pathology of the mitral valve and postoperative LA remodeling, demonstrated a good discriminative power to predict sinus recovery in our patients as well as the other validation populations. This scoring system provides us a treatment policy in the postoperative management. In the low risk population, the anticoagulant protocol maybe modified to decrease the bleeding hazard, especially in the patients who had coagulopathy history such as liver cirrhosis or other comorbidities. However, in the high risk population, this scoring system may remind us to stick to the guideline of anticoagulant recommendations for preventing the embolic event.
